# The effect of inhaling mother’s breast milk odor on the behavioral responses to pain caused by hepatitis B vaccine in preterm infants: a randomized clinical trial

**DOI:** 10.1186/s12887-021-02519-0

**Published:** 2021-02-01

**Authors:** Zahra Akbarian Rad, Parvin Aziznejadroshan, Adeleh Saebi Amiri, Hemmat Gholinia Ahangar, Zahra Valizadehchari

**Affiliations:** 1 Non-Communicable Pediatric Disease Research Center, Health Research Institute, Babol University of Medical Sciences, Babol, I.R Iran; 2 Student Research Committee, Babol University of Medical Sciences, Babol, I.R Iran; 3 Health Research Institute, Babol University of Medicine Sciences, Babol, I.R Iran; 4 Clinical Research development unit of Rohani hospital, Health Research Institute, Babol University of Medical Sciences, Babol, I.R Iran

**Keywords:** Pain, Premature infant, Human milk, Odor, Hepatitis B vaccine

## Abstract

**Background:**

Nowadays, it is generally assumed that non-pharmacologic pain relief in preterm infants is an important measure to consider. Research findings suggest that familiar odors have soothing effects for neonates. The aim of this study was to compare the effect of maternal breast milk odor (MBMO) with that of another mother’s breast milk odor (BMO) on the behavioral responses to pain caused by hepatitis B (HB) vaccine injection in preterm infants.

**Methods:**

This single-blind randomized clinical trial was performed over the period between February 2019 and March 2020 in the neonatal intensive care unit of Babol Rouhani Hospital, Iran. Ninety preterm infants, who were supposed to receive their HB vaccine, were randomly assigned into three groups: MBMO (A), another mother’s BMO (B), and control with distilled water(C). Oxygen saturation (SaO2), blood pressure (BP) and heart rate (HR) were recorded for all participants through electronic monitoring. In addition, premature infant pain profiles (PIPP) were determined through video recording for all three groups during intervention. The chi-square, ANOVA and ANCOVA were used for analyzing the data, and *P* < 0.05 was considered significant in this study.

**Results:**

No significant differences were found between the three groups in mean ± SD of HR, BP, and Sao2 before the intervention (*P* > 0.05). After the intervention, however, the means for heart rate in groups A, B, and C were 146 ± 14.3, 153 ± 17.5 and 155 ± 17.7, respectively (*P* = 0.012). Moreover, the means for PIPP scores in groups A, B and C were 6.6 ± 1.3, 10 ± 2, and 11.4 ± 1.9, respectively (*P* < 0.001). There was no significant difference found between groups in their means of SaO2, systolic and diastolic blood pressure after the intervention (*P* > 0.05).

**Conclusions:**

The results indicate that stimulation with MBMO is effective in reducing pain in preterm infants; therefore, it can be postulated that this technique can be considered in less invasive procedures such as needling.

**Trial registration:**

IRCT, IRCT20190220042771N1. Registered 18 May 2019- Retrospectively registered,

## Background

Many innovative measures to relieve pain in preterm infants are considered by various neonatal intensive care units (NICU) worldwide [1, 2]. It is assumed that neonatal pain in preterm infants can adversely affect their development in such multiple domains as nociceptive changes, altered brain development, stress systems, and functional abilities. Prolonged exposure to pain has also been associated with impaired brain development while preterm infants are in the NICU [3]. Pain assessment methods are currently performed through physiological (heart rate and respiratory rate) and behavioral criteria (crying time, changes in facial expression and limb movements) [4].

The premature infant pain profile (PIPP) is a set of measurable behavioral and physiological responses such as facial expression changes (squeezing eyes, raising eyebrows, wrinkling nasolabial groove) as well as changes in heart rate, SaO2, intrauterine age, and behavioral status of the infants, which are all definite reasons demonstrating pain in premature infants [5].

There is a strong tendency to use non-pharmacological interventions, as simple and secure techniques, for relieving pains in infants. Several methods have already been applied to relieve pain based on five senses [5]. Among them, the sense of smell is fully developed at birth [6] which can affect the neonate’s emotional relationship with his/her mother [7]. Familiar odors, maternal odor for instance, supposedly have soothing effects on newborn infants. It is widely known that infants have the ability to detect their mother’s breast odor even without experiencing breastfeeding at birth [8]. The breast milk odor (BMO) can enhance infants’ sucking through the facial and trigeminal motor nerves in the brain, which, in turn, stabilizes the physiological state in infants [9]. In some cases, research findings has demonstrated that breastfeeding in human newborn infants can completely eliminates pain responses, and animal models have also depicted that the pain modulating effect of breastfeeding is likely mediated by opioid and non-opioid mechanisms [10]. Some studies have shown that Some other studies have also shown that fetal-maternal odors (mother’s breast milk, body and amniotic fluid odors) can decrease stress responses including crying and motor activities in infants, especially those separated from breast milk or the ones under painful interventions [11]. In a relevant study, it was suggested that the maternal breast milk odor (MBMO) had a soothing effect on preterm infants, and that their pain score was lower than that of those exposed to formula odor [12]. Nevertheless, the results of Küçük Alemdar et al. (2017) demonstrated that the BMO made no statistically significant difference in the physiological and behavioral responses of MBMO group compared to other groups (amniotic fluid odor, maternal body odor and control groups) [13].

Given all the contradictory results on the effect of MBMO and another mother’s BMO on preterm infants and the importance of pain relief for preterm infants, this study strove to investigate the effect of inhaling human milk on the behavioral responses of pain caused by HB vaccine in preterm infants.

## Methods

### Study design and setting

This single-blind randomized clinical trial was done from February 2019 to March 2020 in a NICU of academic center (Rouhani Hospital, Babol, Iran).

### Participant

Preterm infants 28–37 weeks of gestation, who have to be vaccinated for hepatitis B –zero turn the vaccine- were randomly assigned to three groups. The inclusion criteria were infants with no painful procedure and no feeding for up to 1 h before the intervention, stability in vital signs, no head and skull abnormalities as well as no receiving painkillers, sedatives and anticonvulsants. The exclusion criteria were maternal withdrawal from the study and infant sever disease or death.

### Groups characteristic

**Fig. 1:**
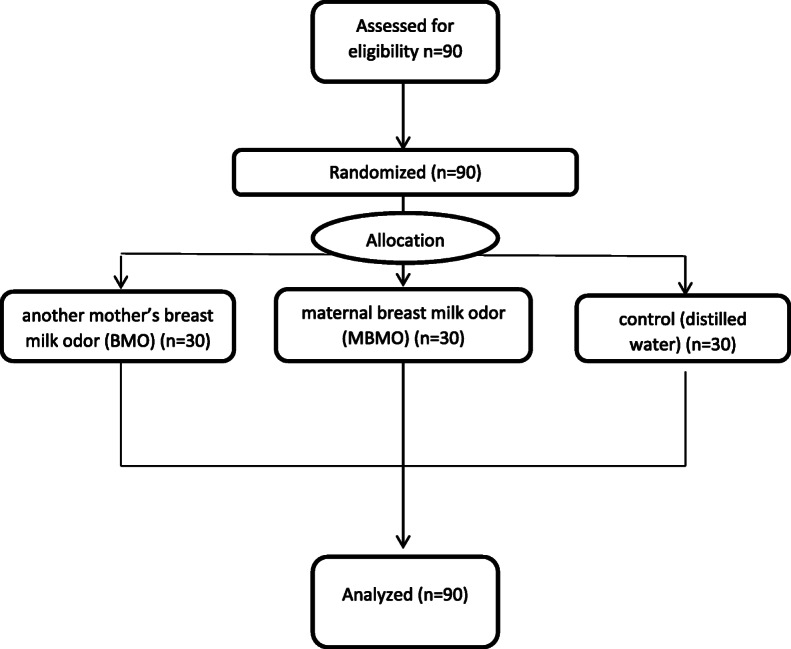
study flowchart: allocation to study groups

After obtaining written consent from the parents, each of the eligible subjects was assigned a number. The numbers were written on paper and tossed into the box, and the desired number was taken out of the box by drawing lots based on the assigned rank. Statistics specialist generated the random allocation sequence, one of the researchers enrolled participants, and assigned infants to three groups: MBMO (A), another mother’s BMO (B) and control with distilled water (C). This study followed the CONSORT guidelines for reporting randomized controlled trials (Fig. 1).

### Sample size

Considering 80% power and 0.05 error probability in this study, the number of cases was determined to be at least 30 neonates in each group [2].

### Data collection and processing

According to the ward’s schedule during the first 4 days of life, injection of HB vaccine was done. Preterm neonate was placed on a warmer by servo control and skin temperature 36.5–37 °C in quiet room. All conditions including room temperature (25 °C), light, injection device were the same for all three groups as well as the vaccine administration was done by one person. The researcher and nurse did not use any aromatic substances in vaccination room during the study. The probe of monitoring was placed on the right wrist of baby without applying additional pressure. Heart rate, blood pressure and SaO2 of all preterm infants were recorded before starting the intervention as the initial time and immediately after completing vaccination (using the standard pulse oximeter and ECG monitoring of Saadat Company, Iran).

In both groups of A and B, the breast milk samples taken in the early morning before eating breakfast were used to stimulate the smell sense of neonates. Pouring 2 ml of maternal breast milk and another mother’s breast milk on a cotton swab was done as an intervention, and 2 ml of distilled water as control group (group C). Next, these swabs were placed three centimeters away from the baby’s nose. This process started 3 min before vaccination and continued until the vaccination was completed [2].

### Pain measurement

The Premature Infant Pain Profile (PIPP) was used as the primary outcome variable. PIPP scores were recorded immediately before and after the vaccination for each infant. The PIPP is a behavioral measure of pain for premature infants. It includes seven indicators: 1) gestational age, 2) the behavioral state, 3) change in heart rate, 4) change in oxygen saturation, 5) brow bulge; 6) eyes squeeze and 7) nasolabial furrow. The total score is the summation of all seven indicators, with a minimum of 0 and maximum of 21; the higher the score, the greater the pain behavior [14]. If the overall PIPP score is between 0 and 6 points, the pain level is mild; if between 7 and 12 points, it is moderate; and if between 13 and 21 points, it is severe [15].

The PIPP tool was revised and validated for use with preterm babies born at 26–37 weeks of gestation by Gibbins et al. in 2014 [16].

Video recording of behavioral responses was taken from the beginning to the end of the process by a trained nurse, and then PIPP scoring was performed through watching video by the first author. The scoring was done while the video viewer was unaware of the test group. Throughout the intervention, any actions on the neonates such as contact, movement and so on were avoided.

Data were collected by using the demographic questionnaire including: birth weight, current disease (respiratory distress syndrome, transient tachypnea of newborn, sepsis and very low birth weight), sex, gestational age, postnatal age, Apgar score and PIPP score.

### Data analysis

Statistics advisor performed the data analysis blindly by using SPSS Version 18. Descriptive information was shown as frequency, percentage, mean and standard deviation. Chi-square test for the relationship between two qualitative variables (demographic and PIPP qualitative variables with group variable), ANOVA test for comparing quantitative variables at the levels of more than two variables (quantitative demographic variables with group variable) and ANCOVA test for comparing research outcomes (SBP, DBP, SaO2 and heart rate) were used to remove the pretest effect and a *P* value< 0.05 were considered significant.

### Ethical consideration

The study protocol was approved by the Ethics Committees of Babol University of Medical Sciences (IR.MUBABOL.REC.1397.253). The trial is registered in the IRCT20190220042771N1 Before participation in the study, written informed consent was obtained from each child’s primary guardian.

## Results

### Study subjects

ALL 90 preterm infants, who included, were completed the study. The infants of the three groups were not significantly different in terms of sex, age, infant’s current disease (Spsis, Respiratory Distress Syndrome (RDS), Transient Tachypnea of Newborn (TTN), very low birth weight (VLBW), gestational age, weight and APGAR score (*p* > 0.05) (Table 1).

**Table 1:** Comparison of demographic variables of preterm infants in three groups

Groups	MBMO(A)	Another mother BMO(B)	Control(C)	*P* value
Variable				
Sex n(%)				0.562^a^
Male	15 (50)	16 (53.3)	12 (40)	
Female	15 (50)	14 (46.7)	18 (60)	
Infant’s age (hour) n(%)				0.112^a^
24–48	16 (53.3)	12 (40)	20 (66.7)	
48–96	14(46.7)	18 (60)	10 (33.3)	
Infant’s disease n(%)				0.943^a^
RDS, Sepsis	3 (10)	4 (13.3)	3 (10)	
VLBW	22 (73.3)	23 (67.7)	23 (67.7)	
TTN	5 (16.7)	3 (10)	4 (13.4)	
Infant’s gestational age (WK) (Mean ± SD)	32.9 ± 2.4	31.5 ± 2.1	32.5 ± 2.4	0.074^b^
Infant’s weight (g) (Mean ± SD)	1806 ± 553	1620 ± 425	1688 ± 404	0.294^b^
Infant’sApgar score(Mean ± SD)	7.8 ± 1	7.6 ± 1.3	8.3 ± 0.9	0.071^b^

^a^chi^2^, ^b^ANOVA

Table 2 shows variables including SBP, DBP, SaO2 and heart rate before and after the intervention by using ANCOVA test.

**Table 2:** Mean and standard deviation scores of SBP, DBP, SaO2 and heart rate in the studied groups pre and post intervention

Groups	MBMO(A)		Another mother’s BMO(B)		Control(C)		***P*** value^a^
Variables	Pre intervention	Post intervention	Pre intervention	Post intervention	Pre intervention	Post intervention	
SBP (mm Hg)	69.3 ± 9.4	70.9 ± 8.2	69.5 ± 7.6	70.2 ± 6	69.7 ± 9	71.7 ± 9	0.482
DBP (mm Hg)	40.6 ± 9.8	43.6 ± 0.5	40.8 ± 9.9	41.7 ± 7.1	40.9 ± 7.9	44 ± 10.7	0.341
SaO2 (%)	97 ± 2.7	95.2 ± 5.2	97.1 ± 3.7	94 ± 6.2	96.4 ± 3.2	91.1 ± 11.7	0.193
Heart rate	139 ± 16.1	146 ± 14.3	141 ± 15.6	153 ± 15.5	139 ± 17.8	155 ± 17.7	0.012

^a^ANCOVA test

As shown in Table 2, by eliminating the effect of the pretest variable and use of ANCOVA test, there is no significant difference between the means± SD of SBP (*p* = 0.482), DBP (*p* = 0.341) and SaO2 (*p* = 0.193) in terms of group membership. ANCOVA test showed that change in heart rate was significantly lower in group A (*p* = 0.012) (Table 2).

### PIPP score

**Fig. 2:**
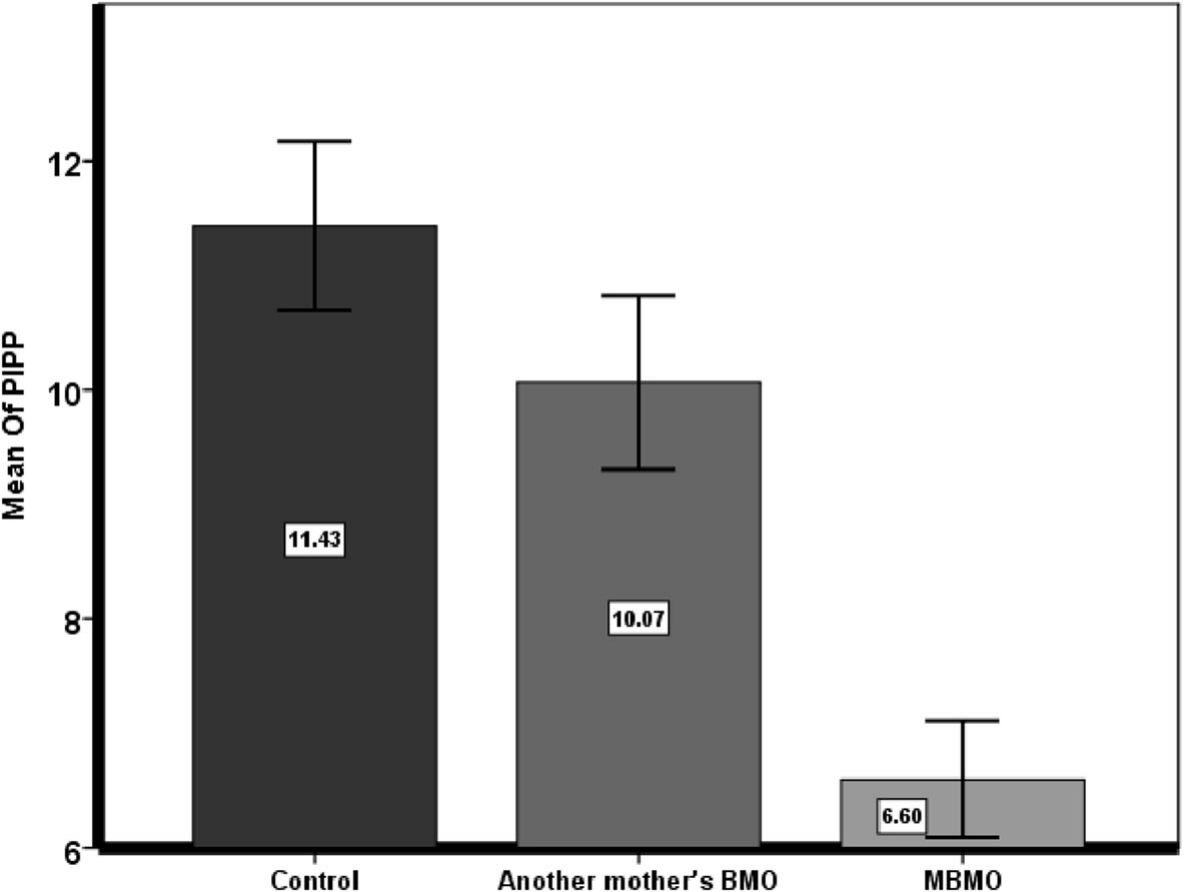
The PIPP score’s changes in three groups (Note: the same letters indicate no significant difference at level 5%)

The mean ± SD of pain score in group A was 6.6 ± 1.3, and 10 ± 2 and 11.4 ± 1.9 in groups B and C, respectively. The ANOVA test showed that there is a significant difference between groups (*P* < 0.001), and the results of post-hoc Tukey’s test determined that this difference was between group A with groups B and C (Fig. 2).

## Discussion

This study showed that MBMO greatly affected heart rate as well as behavioral responses to pain scoring in preterm infants compared with another mother’s BMO and the control groups, but there were no significant differences found between the three groups in terms of SBP, DBP and SaO2.

Zhang et al. (2018) conducted a systematic review study to investigate the analgesic effects of BMO on infants. Their results demonstrated that there was a change in the heart rate of infants and that SaO2 pain scores were lower in MBMO group during and after blood sampling compared with those of the control group [17], It is worth noting that their result is consistent with the findings of the current study, except for SaO2. The stimulation with MBMO had a soothing effect on the behavioral responses to pain and reduced infant’s pain in our study.

In another relevant study, Sajjadi et al. (2016) reported that the mean scores of PIPP had a significant effect on MBMO group compared to control group [2]. Nonetheless, a significant difference was found in the heart rate as well as SaO2 after the intervention. Their results are in line with those of the present study, except for change in SaO2. Likewise, Küçük Alemdar et al. (2017) conducted a study through which they investigated the effect of mother’s BMO, amniotic fluid odor, and body odor on the physiological and behavioral responses to heel stick pain in preterm infants and found no statistically significant difference between groups in terms of physiological and behavioral responses to pain such as heart rate, duration of crying and pain scale. Although the SaO2 was slightly different in the amniotic fluid odor group [13], As it can be seen, this finding is vividly inconsistent with the results of the present study. One possible reason for this discrepancy is the difference between both studies in terms of methodology and intervention process. In their study, 5 cc of the mother’s breast milk was poured on a sponge and placed five centimeters away from the neonate’s nose for fifteen minutes before and after the intervention,, while the cotton swab had been placed three centimeters away from the infant’s nose in our study. This process started 3 min before vaccination in the current study and continued until the vaccination was completed. Attempts were also made to minimize the effect of accustoming to the sense of smell in our study. Aziznejad et al. (2013) evaluated the physiological indicators and concluded that there was a statistically significant difference in the respiratory rate only between the intervention group with sucrose and the other groups immediately after the intervention, but there was no significant difference between the four groups in other variables (duration of crying, heart rate and SaO2) [18].

In three above-mentioned studies (Zhang, Sajjadi and Küçük Alemdar) which were different with our study in terms of methodology, there were no significant changes found in SaO2 between the intervention group and the control group. Moreover, in a similar study by Aziznejad et al. (2013), which was performed under the same condition, there were no differences in SaO2, either. One possible reason could be the difference in the equipment used.

## Conclusion

On the basis of this research, the MBMO can be used as a familiar smell to manage the preterm infant’s pain before performing any needling procedures such as vaccination.

### Limitations

Due to the limited amount of equipment, the use of special probes for infants during the study was provided by several companies. The differences in the sensitivity of these probes may have caused the SaO2 changes not to be accurately determined.

## Data Availability

The datasets analyzed in the current study are not publicly available due to an agreement with the participants upon the confidentiality of the data, but they are available from the corresponding author upon request.

## Abbreviations

ANOVA: Analysis of Variance
MBMO: Maternal Breast Milk Odor
BMO: Another Mother’s Breast Milk Odor
HBV: Hepatitis B Vaccine
HR: Heart Rate
NICU: Neonatal Intensive Care Unit
PIPP: Premature Infant Pain Profile
SBP: Systolic Blood Pressure
DBP: Diastolic Blood Pressure
SaO2: Arterial Oxygen Saturation
RDS: Respiratory Distress Syndrome
VLBW: Very low birth weight
TTN: Transient Tachypnea of Newborn
ANCOVA: Analysis of Covariance
chi^2^: Chi-square
APGAR: Appearance, Pulse, Grimace, Activity, Respiration
